# Comparison between endoscopic and open surgery in 37 patients with nasopharyngeal angiofibroma

**DOI:** 10.1590/S1808-86942012000100012

**Published:** 2015-10-20

**Authors:** José Alberto Alves Oliveira, Marylane Galvão Tavares, Carolina Veras Aguiar, Jorge Ferreira de Azevedo, João Renato F. Sousa, Paulo César de Almeida, Erika Ferreira Gomes

**Affiliations:** a Undergraduate medical student, Ceara State University (Universidade Estadual do Ceará or UECE) (Undergraduate medical student, Ceara State University or UECE); b Medical doctor, residence in otorhinolaryngology, Fortaleza General Hospital (Hospital Geral de Fortaleza or HGF) SESA/SUS (Otorhinolaryngologist, San Dieu Clinic); c Medical doctor, residence in otorhinolaryngology, Fortaleza General Hospital or HGF, SESA/SUS (Otorhinolaryngologist, Otos Clinic); d Master's degree in otorhinolaryngology, Sao Paulo Federal University (Universidade Federal de São Paulo or Unifesp) (Head of the Head & Neck Surgery Unit, Fortaleza General Hospital, SESA/SUS); e Neuroendovascular surgeon (Neuroendovascular surgeon, Fortaleza General Hospital, SESA/SUS); f Doctoral degree in public health, Sao Paulo University (Universidade de São Paulo or USP) (Adjunct professor, Health Science Center (Centro de Ciências da Saúde), Ceara State University or UECE.; g Medical doctor, doctoral degree in otorhinolaryngology, Sao Paulo University or USP. (Tutor of the residency program, Fortaleza General Hospital, SESA/SUS; doctoral student of otorhinolaryngology, Sao Paulo University or USP. Fortaleza General Hospital (Hospital Geral de Fortaleza)

**Keywords:** angiofibroma, neoplasm staging, video-assisted surgery

## Abstract

Juvenile nasopharyngeal angiofibroma is a rare benign vascular tumor of the nasopharynx. Although the treatment of choice is surgery, there is no consensus on what is the best approach.

**Aim:**

To compare surgical time and intraoperative transfusion requirements in patients undergoing endoscopic surgery versus open / combined and relate the need for transfusion during surgery with the time between embolization and surgery.

**Material and Methods:**

Study descriptive, analytical, retrospective study with a quantitative approach developed in the Otorhinolaryngology department of a teaching hospital. Analyzed 37 patients with angiofibroma undergoing surgical treatment. Data obtained from medical records. Analyzed with tests of the Fisher-Freeman-Halton and Games-Howell. Was considered significant if *p* <0.05.

**Study design:**

Historical cohort study with cross-sectional.

**Results:**

The endoscopic approach had a shorter operative time (*p* <0.0001). There is less need for transfusion during surgery when the embolization was performed on the fourth day.

**Conclusion:**

This suggests that the period ahead would be ideal to perform the process of embolization and endoscopic surgery by demanding less time would be associated with a lower morbidity. This study, however, failed to show which group of patients according to tumor stage would benefit from specific technical.

## INTRODUCTION

The juvenile nasopharyngeal angiofibroma is a rare, highly vascular benign tumor of the nasopharynx. It comprises about 0.5% of all head and neck neoplasms. The incidence is 1:150.000 and it affects males in the 14 to 25 years age group^1^.

Accumulation in the nucleus of β-catenin, a co-activator of androgenic receptors, may explain why this tumor selects the male sex. Normal serum levels of hormones in patients with juvenile nasopharyngeal angiofibroma corroborate this explanation^2^.

The typical presentation of juvenile nasopharyngeal angiofibroma consists of chronic nasal block, epistaxis, rhinorrhea, and pain. Because of its invasive growth, it may lead to facial deformity, proptosis, abnormal visual acuity if the orbit is involved, and paralysis of cranial nerves if there is intracranial involvement^2^, ^3^.

A correct diagnosis is made based on the clinical history, the otorhinolaryngologic physical examination, and imaging; these procedures may avoid the need for biopsy, with its risk of hemorrhage. The radiologic exam of choice is contrasted computed tomography of the nose and paranasal sinuses; a tumor in the nasopharynx with widening of the sphenopalatine foramen and marked contrast uptake may be seen. Magnetic resonance imaging is used if there is cranial and orbitary invasion. Digital subtraction selective angiography may be used for the diagnosis and treatment by identifying the blood vessels that feed the tumor and offering the opportunity to embolize these vessels preoperatively^3^, ^4^.

Surgery is the treatment of choice for juvenile nasopharyngeal angio**fibroma; there are also pub**lished descriptions of radiotherapy, hormone therapy, cryotherapy, electrocoagulation, and chemotherapy. Surgery for juvenile nasopharyngeal angiofibroma may be endoscopic, open, or combined^5^, ^6^, ^7^.

The choice of surgery is based on the extent of the tumor and the surgeon's experience. Staging is based on any of the Radkwoski, Chandler, Sessions, Andrews, or Fisch classifications; the latter is used more often^3^.

Endoscopic surgery is a possible treatment approach when tumors are in stages I and II (Fisch) because the morbidity is lower.^6^ It may also be used together with traditional surgery to reduce operative complications and tumor recurrences^8^.

The purpose of this study was to compare the duration of surgery in each approach (open, endoscopic, and combined surgery), and to assess the need for intraoperative blood transfusion compared to the time elapsed since embolization.

## MATERIAL AND METHOD

A retrospective, descriptive, analytic, quantitative, cross-sectional cohort study was carried out at the otorhinolaryngology unit of a tertiary teaching hospital.

The files of patients operated from 2001 to 2010 were reviewed. The sample comprised 37 patients who underwent surgical removal of a nasopharyngeal angiofibroma. The inclusion criteria were a histologic diagnosis of nasal angiofibroma at our institution, and having undergone preoperative embolization with alcohol-polyvinyl 150-350 μm particles.

The study variables were the general characteristics of patients (age and symptoms), of surgical procedures (duration, in minutes; transfusion; embolization; and type of approach), and tumor staging. Surgeries comprised two groups: endoscopic, and open/ combined surgery. The surgical approach across ten years took into account the surgeon's learning curve for endoscopic surgery and the gradual replacement of the open technique.

The Fisch classification was used for staging: stage I (tumor limited to the nasopharynx and nasal cavity with no bone destruction), stage II (tumor invading the pterygomaxillary fossa, the maxillary antrum, the ethmoid and sphenoid sinuses with bone destruction), stage III (tumor invading the infratemporal fossa, the orbit, and the parasellar area, but remaining lateral to the cavernous sinus), and stage IV (tumor with invasion of the cavernous sinus, the optic chiasm or the pituitary fossa)^3^, ^9^.

An analysis of the time elapsed between embolization and surgery was made to identify the day that concentrated most patients (fourth day) and compare it with the remaining days.

The same surgeons carried out all surgical procedures (E.F.G).

The SPSS version 16.0 statistics software was applied for data processing. Simple and percentage frequencies, parametric measures, means and the standard deviation were the basis for data analysis. The Games-Howell test was applied to analyze the difference between mean pairs, as the variances were unequal. The Fisher-Freeman-Halton test was applied for association analyses. The significance level was *p* < 0.05.

## RESULTS

Of 37 patients that underwent surgery for the nasopharyngeal angiofibroma, the mean age was 17 ± 7 years, ranging from 8 years to 44 years. The maxillary artery was the blood vessel that irrigated the tumor in all cases. The ascending pharyngeal and the ophthalmic arteries also contributed to irrigate the tumor in one case, and two direct branches of the internal carotid fed the tumor in two other cases.

The time elapsed between embolization and surgery ranged from 1 to 14 days (mean 5 ± 3 days). The symptoms at the time of diagnosis were: epistaxis (96.8%), nasal block (87.8%), facial bulging (22.5%), nasal discharge (19.4%), ocular symptoms (16.1%), facial pain/headache (12.9%), and hypoacusis (3.2%).

The distribution of patients according to tumor staging was as follows: stage I, one patient (3%); stage II, 23 patients (62%); stage III, nine patients (24%); and stage IV, four patients (11%) (Table 1).

**Table 1:** General characteristics of patients undergoing surgery for the treatment of juvenile nasopharyngeal angiofibroma; January 2001/ July 2010.

Patient	Stage	Technique	Duration of surgery (min)	Preoperative embolization (days)	Transfusion (RBC concentrates)
1	III	Endoscopy+Degloving	360	2	1000 ml
2	II	Endoscopy+Transmaxilar	270	4	No
3	III	Endoscopy+Transmaxillary	200	7	1000 ml
4	IV	Endoscopy+Weber Ferguson	360	5	No
5	IV	Endoscopy+Weber Ferguson	210	3	No
6	IV	Degloving	170	14	No
7	II	Degloving	230	10	No
8	II	Degloving	230	8	No
9	II	Degloving	310	5	1000 ml
10	IV	Degloving	360	3	1000 ml
11	II	Degloving	270	7	500 ml
12	II	Degloving	195	2	500 ml
13	III	Degloving	330	6	No
14	II	Degloving	260	4	No
15	III	Degloving	240	6	1000 ml
16	II	Endoscopy	120	4	No
17	II	Endoscopy	170	2	1000 ml
18	II	Endoscopy	180	3	No
19	II	Endoscopy	150	4	No
20	II	Endoscopy	120	6	No
21	II	Endoscopy	120	7	No
22	I	Endoscopy	80	4	No
23	II	Endoscopy	90	2	No
24	II	Endoscopy	180	4	No
25	II	Endoscopy	120	5	No
26	II	Endoscopy	120	4	No
27	II	Endoscopy	120	4	No
28	II	Endoscopy	150	4	No
29	III	Endoscopy	120	4	No
30	III	Endoscopy	75	3	No
31	II	Endoscopy	205	5	No
32	II	Endoscopy	120	6	No
33	II	Endoscopy	105	1	No
34	II	Endoscopy	100	2	No
35	III	Endoscopy	150	3	No
36	III	Transmaxillary + Transfacial	440	1	4500 ml + 1 RBC concentrate
37	III	Weber Ferguson	195	5	No

Intraoperative transfusions were needed in nine patients. Of these, eight underwent open surgery and one underwent endoscopic surgery (Table 1).

A comparison of patients in which embolization was done four days before surgery (most patients) with patients in which embolization was done on other days revealed that intraoperative transfusions were not required when embolization was done specifically on this day (*p*>0.05) (Table 2).

**Table 2:** Need for intraoperative transfusion versus time since tumor embolization; January 2001 / July 2010.

Transfusion Time (days) * p=0.079										
Yes	1 1 50%	2 3 60%	3 1 25%	**4** **-**	5 1 20%	6 1 25%	7 2 33.3%	8 -	10 -	14 -
No	1 50%	2 40%	4 75%	10*100%	4 80%	3 75%	1 66.6%	1 100%	1 100%	1 100%

* Fisher-Freeman-Halton test

A comparison of the mean duration of surgery among the surgical procedures showed that endoscopy required less time than open/combined surgery (Table 3).

**Table 3:** Comparison of the mean duration of surgery according to each type of procedure; January 2001 / July 2010.

Surgery Duration of surgery (min).			
Type of Surgery	N	%	Mean ± Standard Deviation **p < 0.00012
Open / Combined	17	45.9	272.35 ± 75.93**
Endoscopy	20	54.1	129.75 ± 34.70**

** Games-Howell test

Most of the patients of all tumor stages (except for stage I) that required transfusions during surgery belonged to the open/combined surgery group. The mean duration of surgery was higher in this group compared to the group of patients that underwent endoscopy. There was, however, no significant relationship between tumor stage and the need for intraoperative transfusion (Table 4).

**Table 4:** Association between the type of surgery and tumor staging; January 2001 / July 2010.

Stage / Surgery	Need for transfusion Yes No	Duration of surgery Mean (MIN) ± SD
Stage I Open / Combined (n= 0)	-	-
Endoscopy (n=1)	- 1(100%)	80.00 ± 00.00
Open / Combined (n= 7)	3 (43%) 4(57%) *p=0.061	252.14 ± 37.17 **p<0.0001
Endoscopy (n=16)	1 (6.2%) 15(93.8%)	135.63 ± 33.00
Stage III	2 (33.3%)	294.17 ± 98.51
Open / Combined (n= 6)	4 (66.6%) *p=0.500	**p<0.0001
Endoscopy (n=3)	- 3 (100%)	115.00 ±37.74
Stage IV Open / Combined (n= 4)	1 (25%) 3(75%)	275.00 ± 99.49
Endoscopy (n=0)	– –	–

* Fisher-Freeman-Halton test

** Games-Howell test

## DISCUSSION

Prior embolization of the tumor makes it easier to identify anatomical landmarks during surgery without significant bleeding, thereby facilitating the procedure. Consequently, prior embolization also helps reduce surgical morbidity^6^.

Published papers have shown that the ideal time for embolization of tumors is one to three days before endoscopy^8^. However, our results showed that blood transfusion was less necessary when embolization was done four days before surgery compared to other number of days before; furthermore, surgeons found that the four day time period yielded a better cleavage plane and less perioperative bleeding.

Endoscopic surgery has many advantages compared to the traditional approaches. The main one is that a wider view of the tumor and anatomical landmarks from several angles becomes possible, which yields a better visualization of the interface between the tumor and adjacent soft tissues and bone. The result is a more accurate and complete dissection and better control of bleeding^4^, ^10^. A further advantage of endoscopic surgery is that incisions on the skin and mucosa and facial osteotomies are unnecessary, thereby affecting less the growth of the midface of adolescents. Incisions and closure is not needed, which reduces the duration of surgery and postoperative complications, such as dysesthesia, pain, and trismus, which occur in some of the external approaches^4^.

Yiotakis et al. ^9^ studied the postoperative complications of 20 patients in which a nasopharyngeal angiofibroma was removed and concluded that the endoscopic approach after embolization is safe, effective, and associated with fewer complications; it is thus ideal for tumors at initial stages. In our series, the mean duration of surgery for endoscopy was 107.7 minutes; for open procedures it was over 200 minutes (203 minutes for degloving, and 270 minutes for transpalatine surgery). Our findings corroborate these findings; our mean duration for endoscopy was 130 minutes, and for open surgery it was 270 minutes, suggesting that the latter could cause more morbidity.

Among our patients, only one of nine patients that required blood transfusion had undergone endoscopic surgery. We found no relationship as to which would be the best technique for a given stage when analyzing the need for blood transfusions and tumor staging; the patients were distributed non-homogeneously within both surgery groups.

A relatively low blood loss during endoscopic procedures may occur due to the careful nature of dissection. Even small amounts of bleeding limit the surgeon's endoscopic view, so special attention should be given to hemostasis for satisfactory results. Furthermore, most of the blood loss in open approaches occurs because of incisions and osteotomies for surgical access^5^.

Pryor et al.^5^ studied 54 patients in which nasopharyngeal angiofibromas were removed; open surgery was done in 49 patients and endoscopic surgery was done in five patients. The authors found that there was less intraoperative bleeding, a shorter hospital stay, and fewer complications and recurrences in patients that underwent endoscopic surgery. The authors also suggested that tumors involving the ethmoid, the maxillary sinus, the sphenoid sinus, and stage I and II (Fisch) can be operate by endoscopy. Tumors that involve the orbit or the cavernous sinus require an intra- and extracranial approach.

Midfacial degloving is a reasonable choice for nearly all advanced tumors – Fisch's stage IV – as it is useful for providing a good surgery view during surgery, esthetic results, and lower morbidity; if necessary, it may be combined with other approaches or craniotomy^11^, ^12^.

The contraindications against endoscopy are stage IV and some stage III nasopharyngeal angiofibromas, which extend to the mid-cranial fossa. It is advantageous to combine midfacial degloving with endoscopy when removing tumors that extend through the anterior portion of the cribbous lamina, and also to eventually correct cerebrospinal fluid leaks^13^.

Extension of the tumor to the lateral infratemporal fossa, the sellar area, and areas adjacent to the optic nerve is a challenge for endoscopic resection. When tumors invade these areas, the surgical view is limited even in open procedures and tumors are hard to resect. Endoscopy provides lighting, amplification, and multiangle visualization, which may facilitate removal of tumors that are adjacent to these vital areas. Associating open surgery with endoscopy is advantageous in this context, as the benefits of both approaches are explored, and the possibility of tumor recurrence is reduced^8^, ^14^.

Still, Carrau et al.^15^ noted that tumors may be treated by endoscopic surgery alone when the pterygopalatine and infratemporal fossae are involved.

This comparison of our findings with the literature aimed to foster debates on this topic. There is no consensus on when to carry out embolism or which is the best surgical approach for each tumor stage.

## CONCLUSION

We found that the mean duration of surgery was shorter when endoscopy was the chosen technique compared to open approaches, which is possibly due to lower morbidity of endoscopy. There was less need for blood transfusion in patients that underwent embolization four days prior to surgery.
